# Methods to localize cell specific alterations within the 3D context of the lung

**DOI:** 10.3389/fphys.2026.1805715

**Published:** 2026-07-15

**Authors:** Jonas Labode, Christian Mühlfeld

**Affiliations:** 1 Institute of Functional and Applied Anatomy, Hannover Medical School, Hannover, Germany; 2 Biomedical Research in Endstage and Obstructive Lung Disease Hannover (BREATH), Member of the German Center for Lung Research (DZL), Hannover, Germany; 3 Research Core Unit Electron Microscopy, Hannover Medical School, Hannover, Germany

**Keywords:** apoptosis, branching analysis, bronchopulmonary dysplasia, immunohistochemistry, light microscopy, micro-computed tomography

## Abstract

The lung is a heterogeneous organ, composed of morphologically different sub-compartments. When analyzing lung pathologies, it is important to collect the spatial location of lesions, to allow the translation of e.g. preclinical study results to the human lung, or the formulation of targeted treatment strategies. For the purpose of this review, bronchopulmonary dysplasia will serve as an example for a lung pathology to be studied, but the methods demonstrated can be adapted to other pathologies as well. The review’s focus will lie on common histological methods and the potential to use them for both the study of cellular features and location assessment. Morphological characteristics, as well as intracellular states related to cell cycle and cell death will be considered. In an advanced section, the combination of multiple imaging methods into analysis pipelines is detailed, describing different combinatorial possibilities with their advantages and use in the research context. Through the integration of microscopy and computed tomography into one process, it is possible to assess both 3D location and cellular features, with the option of employing immunohistochemical markers for the detection of specific proteins as well. This is a tool that can be used in addressing both transitional and longitudinal research questions regarding lung pathologies.

## 1 Introduction

Mammalian lungs have a highly heterogeneous architecture. The airways form a tree-like structure, branching out from the trachea down to the alveoli. During this course, they change in overall size, cellular composition and dimensions of the airway epithelium, as well as number of smooth muscle cell layers and presence of glands or supporting cartilage. The terminal bronchioles then mark the change from an air-conductive system, optimized for airflow, to a compartment optimized for gas diffusion. A similarly diverse structure is found in the vasculature, where dimensions and composition differ greatly between e.g. central pulmonary artery and the capillaries enveloping the alveoli (Weibel, 2009).

This multi-faceted organ composition leads to different levels of susceptibility to external stressors, implications of damage and presentation of lesions between sub-compartments of differing morpho-functional composition. It is therefore essential for the use of preclinical animal models of lung diseases, to determine the location of observed changes, both for the facilitation of adequate comparisons between e.g. treatment and control group, as well as for the translation of the results to the human lung. This article will focus on Bronchopulmonary dysplasia (BPD) as a source of lung damage, but the methods listed here are applicable to other lung pathologies as well.

BPD is a syndrome that affects infants born at an extremely preterm time point (< 28 weeks of gestation), when the lung is still in early stages of development, particularly the canalicular or early saccular phase. Necessary treatments such as mechanical ventilation and hyperoxia cause lung injury, potentially triggering a disruption in alveolarization and microvascular development (Thebaud et al., 2019).

On an organ level, the affected lung may present features such as large airway obstruction and small airway hyperinflation (Bush et al., 2023), enlarged and simplified alveoli (Coalson, 2003), as well as simplification or arrested growth of the pulmonary vasculature (Abman,2008).

On a cellular level, the airways can exhibit morphological alterations like epithelial thickening (Tiddens et al., 2008), ciliary abnormalities (Lee and O’Brodovich, 1988) or an increased number of goblet cells and smooth muscle cells (Hislop and Haworth, 1989). Smooth muscle cell proliferation is also a problem in the vasculature, where it can lead to pulmonary hypertension (Hansmann et al.,2020).

The main driver for these changes is oxidative stress, for which the immature lung is especially susceptible, eventually leading to inflammation and programmed cell death (Kaarteenaho-Wiik and Kinnula, 2004; Wang and Dong, 2018). The premature infant’s antioxidant enzyme system is still underdeveloped. Hyperoxia increases reactive oxygen species (ROS) production in mitochondria and subsequent cellular buildup that causes stress and injury, potentially ending in apoptosis (Deng et al., 2022). ROS buildup can also stimulate increased mucus secretion and pulmonary vasoconstriction (Barnes (1990); Wright et al., 1994).

These changes are not evenly distributed throughout the lung. In a mouse model of hyperoxia, immunohistochemical detection of the tumor suppressor gene p53 showed its accumulation in the distal bronchial epithelial cells, as well as, to a lesser degree, in the alveolar epithelium. This demonstrates a local peak in reaction to oxidative stress, in the form of DNA-damage and subsequent cell cycle arrest, possibly leading to cell senescence or apoptosis as a result (O’Reilly et al.,1998).

Morphological studies on the pulmonary vasculature (Labode et al., 2023) and the airways (Pfleger et al., 2025) in a rabbit model of BPD were also able to demonstrate a heterogeneous distribution of differences between experimental- and control group. Affected sites could be attributed to specific levels of airway branching, displaying a diverse pathology that is not easily captured using purely global methods (i.e. when not recording or taking into account the site of the structure sampled).

The goal of this review is to present different methods for the identification of cellular alterations in the lung and their capabilities for providing spatial context for those loci of interest within the organ’s architecture. We will further present a novel extension for a multi-step imaging approach first established by Grothausmann et al. (2021), demonstrating the possibility of the location focused assessment of intracellular substance levels. The methods discussed here are aimed at preclinical small animal models. Application to human lung pathologies would require modifications to the presented workflows.

## 2 Light microscopy

### 2.1 Histological sections

**Figure 1:**
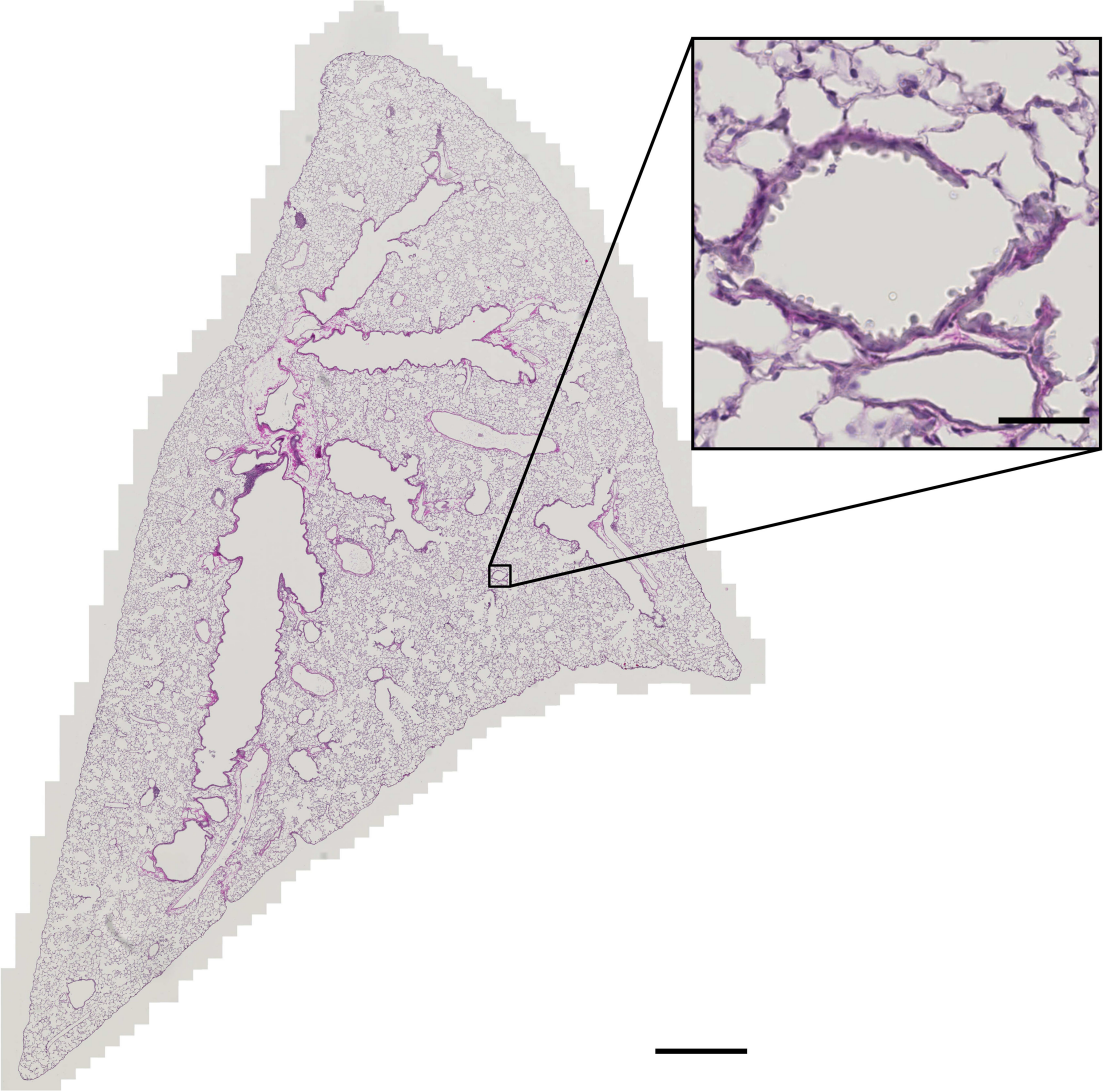
HE stained tissue section from a left mouse lung, image acquired using LM. Scale bar = 1000 µm. Inset: Airway cross section (center), blood vessel below. Within the airway, club cells can be identified by their characteristic bulging into the luminal space. Scale bar = 50 µm.

The morphological study of histological lung sections through a light microscope is a central method in lung research (see e.g. Weibel, 1963; Ochs and Muhlfeld, 2013). It requires little expensive equipment and sample preparations can be performed in most laboratories. A wide range of staining protocols is available for marking features of interest. In preclinical models, that study small animals such as mice or rabbits, lungs can be harvested, embedded and whole lung slices can be placed on one microscopic glass slide to provide 2D spatial context. The achievable magnification of this technique is sufficient to study sub-cellular features (see e.g. Muhlfeld et al.,2021). An example is provided in **Figure 1**, where both a complete organ slice of a mouse lung, as well as a small airway cross section demonstrate the capabilities of this method.

Electron microscopy is able to achieve a significantly higher resolution, aiding the identification of intracellular changes, but in turn sacrifices field of view, i.e. spatial context (Ochs et al., 2016). Therefore, this method will not be discussed here any further.

Histological sections, even when only treated with standard stains, such as hematoxylin and eosin (HE) or toluidine blue, allow the collection of a wide variety of parameters relevant for identifying and quantifying pathological alterations. Examples include volumes and surface areas of various structures, epithelial changes, increases in smooth muscle mass or presence of fibrosis or inflammation. The use of a stereological counting scheme assures unbiased results (Muhlfeld and Ochs, 2013). Late-stage apoptosis can also be identified morphologically, by features such as membrane blebbing, nuclear condensation and formation of apoptotic vesicles (Huppertz et al.,1999; Kumaraswamy et al., 2013). Care must however be taken during the organ fixation. As the lung is a highly dynamic organ, changing its shape during the breathing cycle, its morphology is highly dependent on the pressures applied to it. Thus, fixation must be performed under a physiological pressure setting, as pressures outside that range would lead to unrealistic organ presentations. Well-tuned setups however are able to fix the lung in specific physiological (e.g. inspiration or expiration) or pathophysiological inflation states (Perlman et al., 2024).

For intracellular processes, such as cell cycle arrest or early stages of apoptosis, immunohistochemical markers can be used to visualize involved proteins (Barranco et al., 2011; Xiao et al., 2015). An example would be activated caspase 3, that is present in cells undergoing apoptosis and its visualization can be used for quantification of affected cells (Duan et al., 2003).

**Figure 2:**
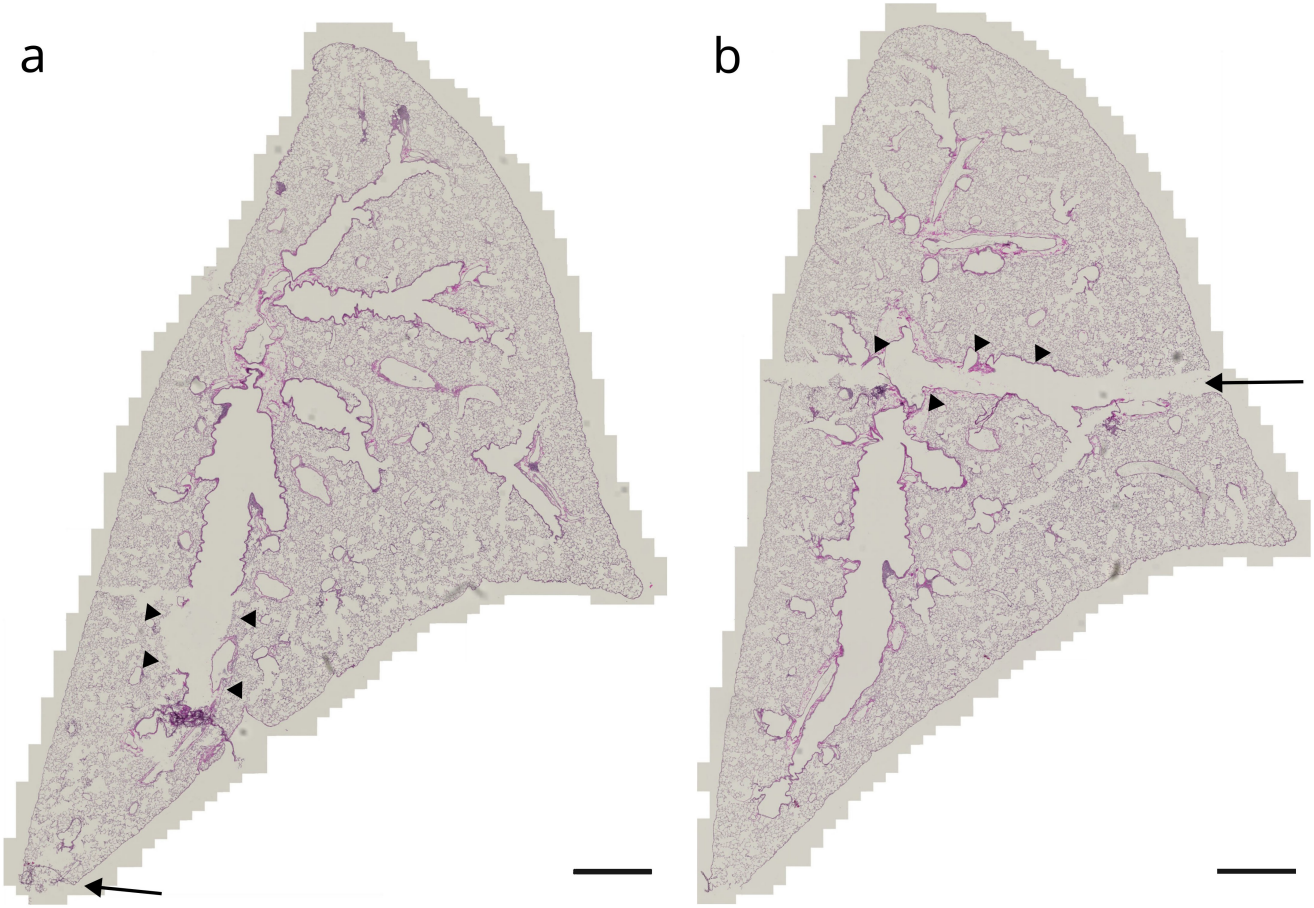
Examples for damages encountered in the cutting process, images acquired using LM. **(a)** Airway wall delamination (arrowheads) and slight folding (lower most tip, arrow). **(b)** Horizontal tear (arrow) through multiple airway cross sections (arrowheads). Scale bars = 1000 µm.

The inherent problem of LM however is, that single histological sections provide no 3D context. An airway cross-section in a LM image is not easily attributed to one specific branching order. The path through the organ, that connects this airway section to trachea and alveoli, is unknown. See e.g. the airway magnified in **Figure 1**. An approach to use LM to create this missing 3D context would be to produce serial sections of histological slices from the organ. This introduces a problem, as the tissue slice orientations on the glass slides will vary. Furthermore, the tissue might be stretched or compressed in the cutting process. To be able to stack them on top of one another to create a 3D representation of the organ, these inter slice differences would need to be removed. The solution to this problem is image registration (see e.g. Hill et al., 2001). Rigid registration algorithms will laterally move and rotate the tissue sections, while non-rigid algorithms will correct deformations. Slices can however be disturbed in the cutting and handling process in additional ways, that registration is unable to correct for adequately. Examples include tears, folds, major deformations, insufficient staining or complete slice loss. While there are mitigation strategies to limit the amount of deformations introduced during preparation (see e.g. Taqi et al., 2018), they are a common problem in computational histology (Kanwal et al., 2022). These artifacts can add up in the registration process and incrementally distort the images beyond usability. Thus, previous studies were only successful in reconstructing small stacks of histological slices (Grothausmann et al., 2017). **Figure 2** exemplifies such damages. In comparison with other organs, the sparse tissue content of the lung seems to offer little structural integrity to withstand the shear forces encountered in the cutting process. Besides these problems, the amount of work to produce an image stack of a whole lung in a resolution high enough to study sub-cellular characteristics should be considered. At the usual 1 µm to 5 µm slice thickness, even an organ in the size range of a few millimeters will create hundreds of slices when completely sampled. Collecting only subsets instead, would result in losing the spatial context, i.e. connection between structures visible in the individual slice sub-stacks.

### 2.2 Light sheet fluorescence microscopy

**Figure 3:**
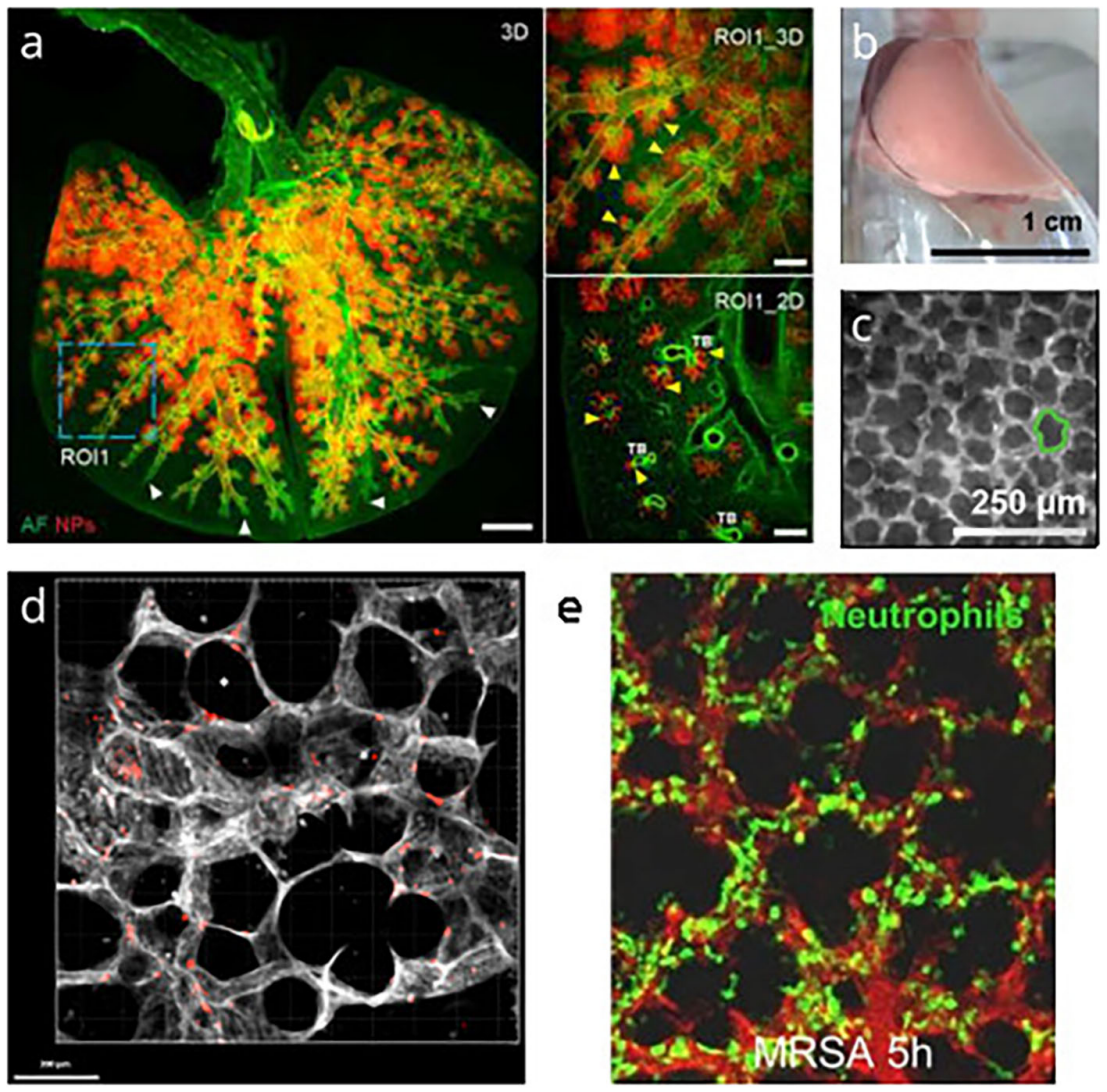
Images produced in different microscopic settings. **(a)** LSFM of a mouse lung, airways green, inhaled nanoparticles red, scale bar main image = 1000 µm, scale bars ROIs = 400 µm (Yang et al. (2023)). **(b)** Mouse lung placed in a crystal ribcage. **(c)** Image captured through crystal ribcage, green marking indicates a region of interest (b, c: LeBourdais et al. (2024)). **(d)** Human PCLS, infected with Legionella pneumophilia marked in red, scale bar = 200 µm (Viana et al. (2021)). **(e)** 2-photon microscopy image of ongoing infection acquired through an intravital window, neutrophils green, vasculature red (Alizadeh-Tabrizi et al. (2021)). All files provided under the Creative Commons license 4.0. Reproduced Yang et al. (2023), CC BY 4.0 License, https://doi.org/10.21203/rs.3.rs-2994336/v1; LeBourdais et al. (2024), CC BY 4.0, http://dx.doi.org/10.3389/fnetp.2024.1396593; Viana et al. (2021), CC BY 4.0, https://doi.org/10.1111/mmi.14817, Alizadeh-Tabrizi et al. (2021), CC BY 4.0, http://dx.doi.org/10.3389/fcell.2020.620471.

A method that is capable of generating microscopic 3D datasets is light sheet fluorescence microscopy (LSFM). In this technique, only one plane of a volumetric specimen is illuminated by a sheet of light. Fluorescent dyes in the illuminated plane can thus be excited and captured by a microscope as a 2D image. By either moving the sample or the light source, the sheet of light can be moved. In this fashion, a series of 2D images along one axis of the sample can be collected and merged into a 3D image volume. *In-vivo* imaging is possible in small organisms (e.g. zebrafish, mouse embryos), but already at mouse size, only single explanted organs can be imaged at high resolution. The limiting factor here is the light scattering by the illuminated tissue. Size limitations can however be overcome to some extend by e.g. chemically clearing the tissue (see Huisken et al., 2004; Daetwyler and Fiolka, 2023). In lung research, this technique has been successfully employed e.g. by Yang et al. (2019) for mapping of the distribution of inhaled nanoparticles along the airways in an explanted mouse lung (see **Figure 3a**). While this method provides high magnification 3D data, the relianc.

### 2.3 Live tissue imaging

The methods detailed above suffer from one major limitation, that is, they are mostly limited to imaging a fixed tissue state, i.e. the cellular configuration of the organ at the time of fixation and embedding. For the study of e.g. pathogenesis and treatment response in real time, it is therefore either necessary to study living lung tissue in an ex-vivo setting or to perform the imaging *in-vivo*.

The oldest technique for investigations on live tissue in this context, is the use of precision cut lung slices (PCLS). For this method, an organ such as a fresh mouse lung is explanted, filled with agarose for stability and then cut into slices on a vibrating microtome. For larger animals or human tissue, biopsies are used instead of full organ cross sections. The slices, usually ranging between 100 µm to 500 µm in thickness, are then placed in a cell culture medium in well plates and can be studied for up to 14 days. In this state, there is no respiration or circulation present. However, there is still a plethora of processes that can be observed. Examples include airway contraction or relaxation as well as vasoconstriction and dilation, mucus secretion and ciliary motion, immune cell activation and mediator release or extracellular matrix deposition or degradation (see e.g. Koziol-White et al., 2024; Liu et al., 2019; Viana et al., 2021). The tissue cultures can be imaged using common microscopic techniques such as bright field or confocal microscopy and fluorescent imaging (see below). Live cell staining protocols are available (see e.g. Akram et al., 2019; Lyons-Cohen et al., 2018)) for tracking e.g. cellular migration in real time. However, often PCLSs will be fixed and embedded in a conventional manner at a specific time point after a treatment or experimental condition was administered. See e.g. van Rijt et al., 2015 who quantified apoptotic tumor cells after administering a drug using a targeted delivery technique. Given the slice thickness, PCLS provide minimal 3D information (see **Figure 3d**), but just as with conventional histological sections, to produce a full 3D organ representation, consecutive sections would need to be registered to one another. This may be further complicated by structural changes during cutting and the subsequent incubation period. So far, the authors are not aware of any publications reporting on the attempt of such a process.

A method that overcomes limitations of PCLS, such as the lack of circulation and breathing motion, as well the missing option for a realistic treatment delivery (i.e. by inhalation or through the bloodstream), is the crystal ribcage. It consists of a mouse lung that is placed in a transparent model of a ribcage and is externally perfused and ventilated (see **Figure 3b**). The transparent housing permits optical imaging of most of the lung’s surface. Imaging depth ranges between 100 µm in air-filled alveoli and 500 µm in areas with reduced light scattering, such as edematous alveoli (see **Figure 3c**). This method excels at visualizing e.g. vascular transport of immune cells or alveolar cells in a sub-cellular resolution during simulated or induced physiological and pathophysiological states (see Banerji et al., 2023). Examples of its use include the assessment of inhaled particle deposition using fluorescence imaging Grifno et al. (2025) or the change of mechanical lung properties in cancer LeBourdais et al., 2024). However, the image acquisition is limited to the organ’s surface and deeper structures are not visible. Grifno et al. (2025) for example used PCLS after aerosol delivery in the crystal ribcage, to assess the role of the conducting airways in the particle deposition.

The previous methods all used explanted lungs, creating an experimental setting that differs in various ways from the *in-vivo* situation. Intravital microscopy on the other hand captures microscopic images of an organ *in-situ*, preserving most of the natural organ surroundings. To achieve this, a window up to one centimeter in diameter is implanted into a mouse at a location in the thorax, where some ribs have been removed. The animal is anesthetized and ventilated during the procedure. Imaging can then be performed through the window in a similar fashion to the crystal ribcage, with the difference of a very limited field of view and a narrow time window for imaging, due to the ongoing narcosis (up to 3 hours have been reported). Imaging is further restricted to subpleural structures with a maximum depth of up to 125 µm (see **Figure 3e**). A main target for this method is imaging of the vasculature, targeting e.g. hemoglobin and oxygen saturation or immune dynamics in the pulmonary microvasculature (see e.g. Hanna et al., 2013; Cleary et al., 2024; Looney et al., 2010). This method offers unique possibilities for the assessment of intravital processes, such as the recruitment of lymphatic cells from other organs to the lung in an inflammatory response. Its basic imaging limitations however are identical to those of the crystal ribcage with the addition of a greatly reduced field of view.

## 3 Micro computed tomography

**Figure 4:**
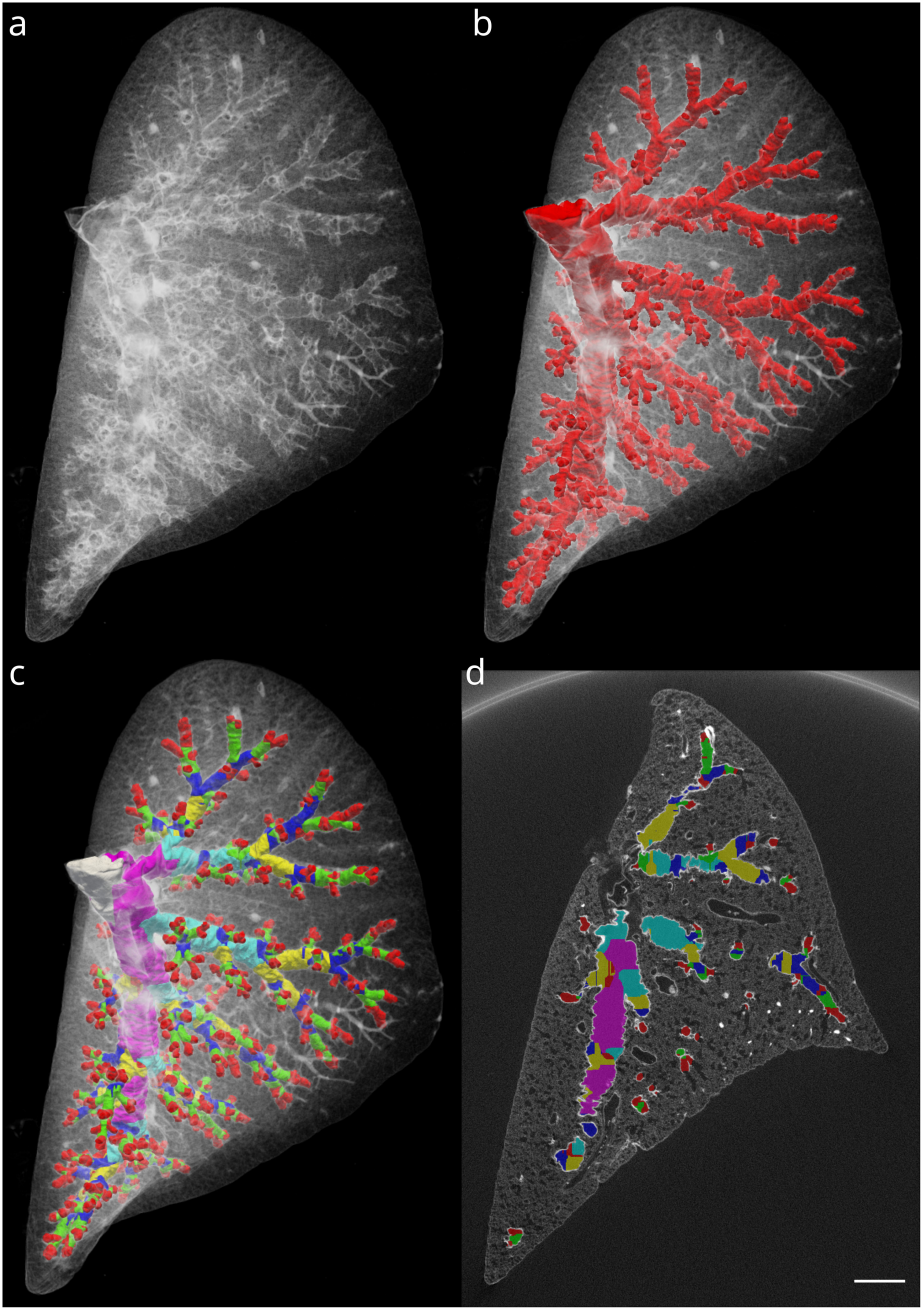
Full µCT volume image of the left mouse lung from **Figure 1**. **(a)** 3D volume rendering of a complete organ µCT scan stained with PTA. **(b)** Same as **(a)**, but including a segmentation of the conducting airways. **(c)** Same as **(b)**, but with the segmentation color coded for Strahler orders. **(d)** 2D image slice from the volume with Strahler order markings. See **Figure 5** for details. Scale bar = 1000 µm. Strahler orders, counting from distal to proximal: Red = 1, green = 2, blue = 3, yellow = 4, cyan = 5, magenta = 6, white = 7.

With the complications of creating a 3D image volume from 2D sections detailed above or the limited imaging depth in the case of surface imaging, an alternative imaging technology that natively supports 3D imaging, like the micro computed tomography (µCT), seems advantageous. It facilitates the destruction-free imaging of complete organs (see e.g. Schambach et al. (2010)). Tissue can be treated with e.g. osmium tetroxide (*OsO*_4_) or phosphotungstic acid (PTA) to achieve the contrast levels necessary for analysis (Metscher, 2009; Quintarelli et al., 1971). See **Figure 4a** for a volume rendering of the µCT scan of a left mouse lung stained with PTA.

The image stack resulting from a µCT scan can be segmented to create models of structures of interest, e.g. the airway-tree (see **Figure 4b**) or pulmonary blood vessels. Available methods are manual tracing of the structures (using e.g. Kremer et al., 1996), semi-automated approaches using threshold algorithms like watersheds (Meyer and Beucher, 1990) in combination with manual interaction (Grothausmann, 2015) or fully automated workflows, employing deep learning methods (Ronneberger et al., 2015; Isensee et al., 2020). The latter method was used for the images presented here. A neural network specifically trained for the segmentation of the conducting airways was used. This model stems from a research project focused on this morphological structure and was chosen for demonstration purposes here, as the resulting segmentation contains most of the airway branching in the lung and terminates at a consistent morphological border, i.e. the transition from bronchial epithelium to airway epithelium.

The resulting segmentation images can then be analyzed for branching patterns and color coded accordingly. Different branching metrics have been established, e.g. standard generations (Weibel, 1963) or orders (Horsfield, 1984), schemes more suited to branching with a high degree of asymmetry (Strahler, 1957) or approaches better matching e.g. particle deposition patterns in the airways (Wang and Kraman, 2004). Also, individual markings for each airway segment can be employed to facilitate data collection on a branch segment level. For the purpose of this work, the Strahler order was applied (see **Figures 4c,d**). It attributes order 1 to the most distal segments of the airway tree and then moves from the periphery to the central structures. If two equal orders intersect, the order is increased for the next segment. If two uneven orders meet, the higher order is kept. This scheme is especially suitable for the asymmetrical branching pattern of monopodial lungs and produces orders of high intra-order morphological similarity (see Labode et al., 2022).

While the methods listed above are applicable for any structure with a tree-like branching pattern, airways can also be classified by anatomical nomenclature (Thoracic Society, 1950). Work has been conducted on adapting this nomenclature to the mouse lung and branching pattern phenotypes in different mouse breeds have been identified using µCT scans (Thiesse et al., 2010). This approach has its advantages in assessing variance in branching patterns and the comparison of individual central airway branches. For the task of identifying morphologically homogeneous groups of airway segments, it performs analogous to the standard generation approach.

Overlayed on the µCT image stack, the color-coded segmentation images can serve as a location indicator for adjacent structures that are analyzed, like the walls of airways or pulmonary vasculature (Labode et al., 2023). In addition, the morphology of the segmented structure itself can be analyzed for parameters such as total volume, number of bifurcations and segments, individual segment length (Hemandez-Cerdan, 2021) or local diameters (Dougherty and Kunzelmann, 2007).

**Figure 5:**
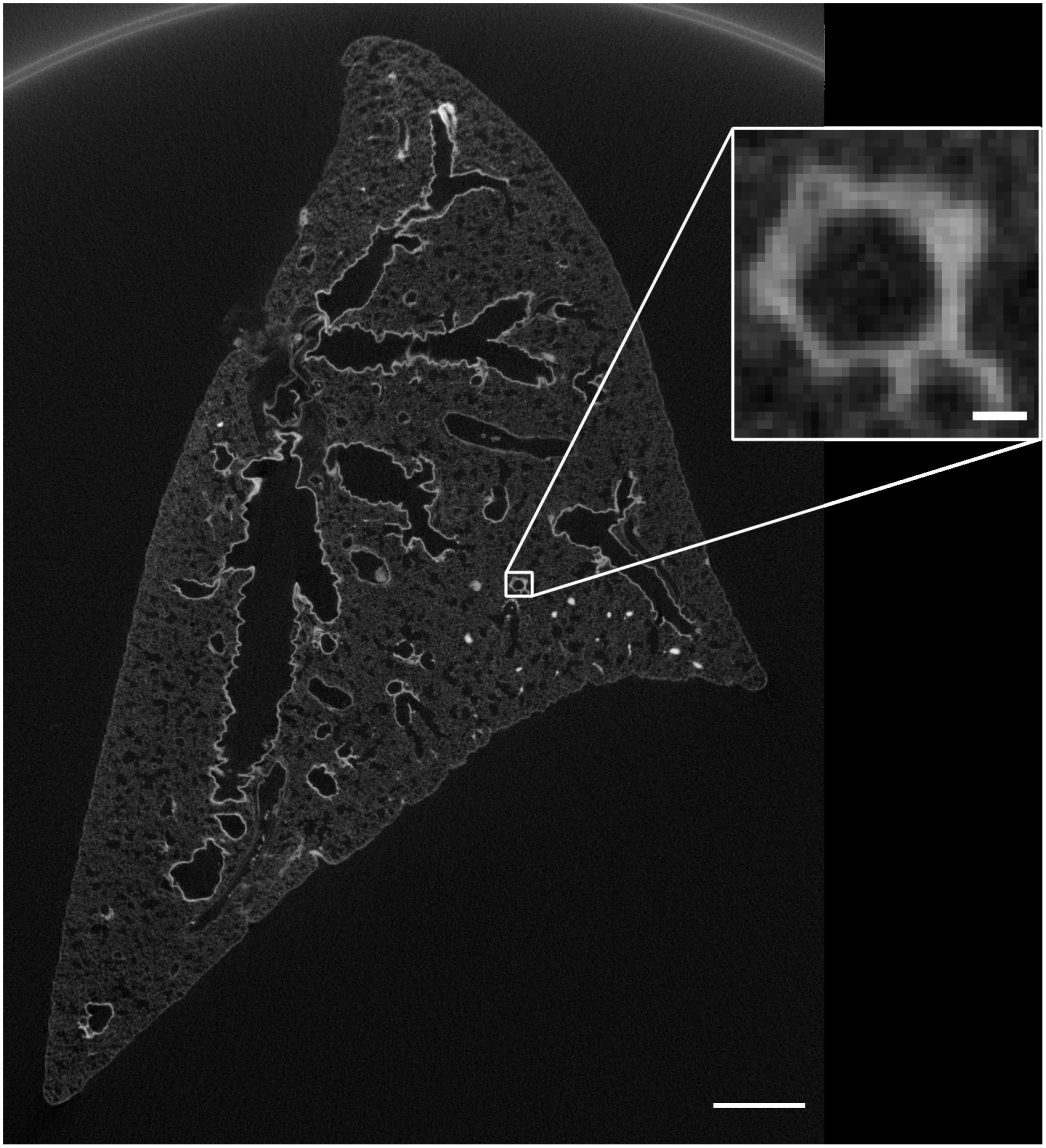
PTA-stained tissue section roughly equal to the location of **Figure 1**, imaged using µCT. Discrepancies between the images are caused by the difference in physical cutting angle and the orientation of the organ within the µCT volume. The white lines in the top corners are imaging artifacts caused by the edge of the surrounding paraffin block. Scale bar = 1000 µm. Inset: Airway cross section (center). While the border of the airway is identifiable, its cellular composition is beyond reach. Scale bar = 50 µm.

The main drawback of µCT is the limited achievable resolution compared to light microscopy. There is a trade-off between field of view and resolution. While nanometer resolutions can be achieved for small tissue sections, *in-vitro* imaging of small animal organs delivers voxel sizes between about 5 µm and 10 µm (Badea et al., 2008). Even when using an advanced synchrotron radiation µCT (SRµCT), the resolution of a mouse lung scan does not exceed 4.4 µm voxel size, partially sacrificing some distal tissue sections in the process (Labode et al., 2022). Thus, features that can be assessed are only the coarse morphology, e.g. airway diameter or wall thickness. Cellular surface differentiation, like ciliary abnormalities or apoptotic cell blebbing lie far beyond reach. This limits the use in research questions focused on changes in the gas exchange regions. The observation of e.g. alveologenesis, fibrotic remodeling or inflammatory processes is limited to major morphological changes rather than the underlying cellular mechanics. See **Figure 5** for a demonstration of how the practical capabilities of the µCT differ from those of the LM, as displayed in **Figure 1**. For the segmentation of the airways this implies, that the most distal part that can be segmented in a reliable fashion are the ductuli alveolares. The septa between single alveoli however are not resolved well enough to perform a complete segmentation of the gas exchange region. Attribution of features within that area to a specific airway could only be performed heuristically, e.g. by a nearest neighbor classifier (see e.g. Hastie et al.,2009).

The ability to mark specific structures, that is available in LM images using e.g. multi-colored stains, is very limited in the monochromatic µCT images. Options include the pairing of e.g. gold nanoparticles with antibodies to be used as markers in preclinical and clinical CT imaging (Hsu et al., 2020; Ashton et al., 2015). Due to the particle size, these are only able to bind to the cellular surface, but do not penetrate inside. Therefore, they are unusable for visualizing e.g. the apoptotic signaling cascade within the cell. Nevertheless, they can be used for marking inflammatory reactions (Wyss et al., 2009).

For intracellular processes like apoptosis, radiotracers are available, but these require positron emission tomography to visualize. They are used e.g. for assessing chemotherapy results in cancer patients (Beroske et al., 2021); Rybczynska et al., 2018) and can also be combined with ex-vivo imaging (Maris et al., 2024). Other than that, only the specific affinity of different contrast agents for distinct tissue types can be used to allow the visualization of cellular characteristics in µCT scans. See Keklikoglou et al. (2019) for list of common contrast agents and their tissue staining properties.

## 4 Multi step imaging pipelines

### 4.1 Established workflows

Due to the limitations of the imaging methods listed above, there have been efforts to combine multiple imaging techniques into one analysis workflow to profit from their individual advantages.

Grothausmann et al. (2021) devised a method for registering µCT data and corresponding LM images to one another, so that the branching information from a segmentation of a µCT volume image can be incorporated into high resolution LM images to provide spatial context.

**Figure 6:**
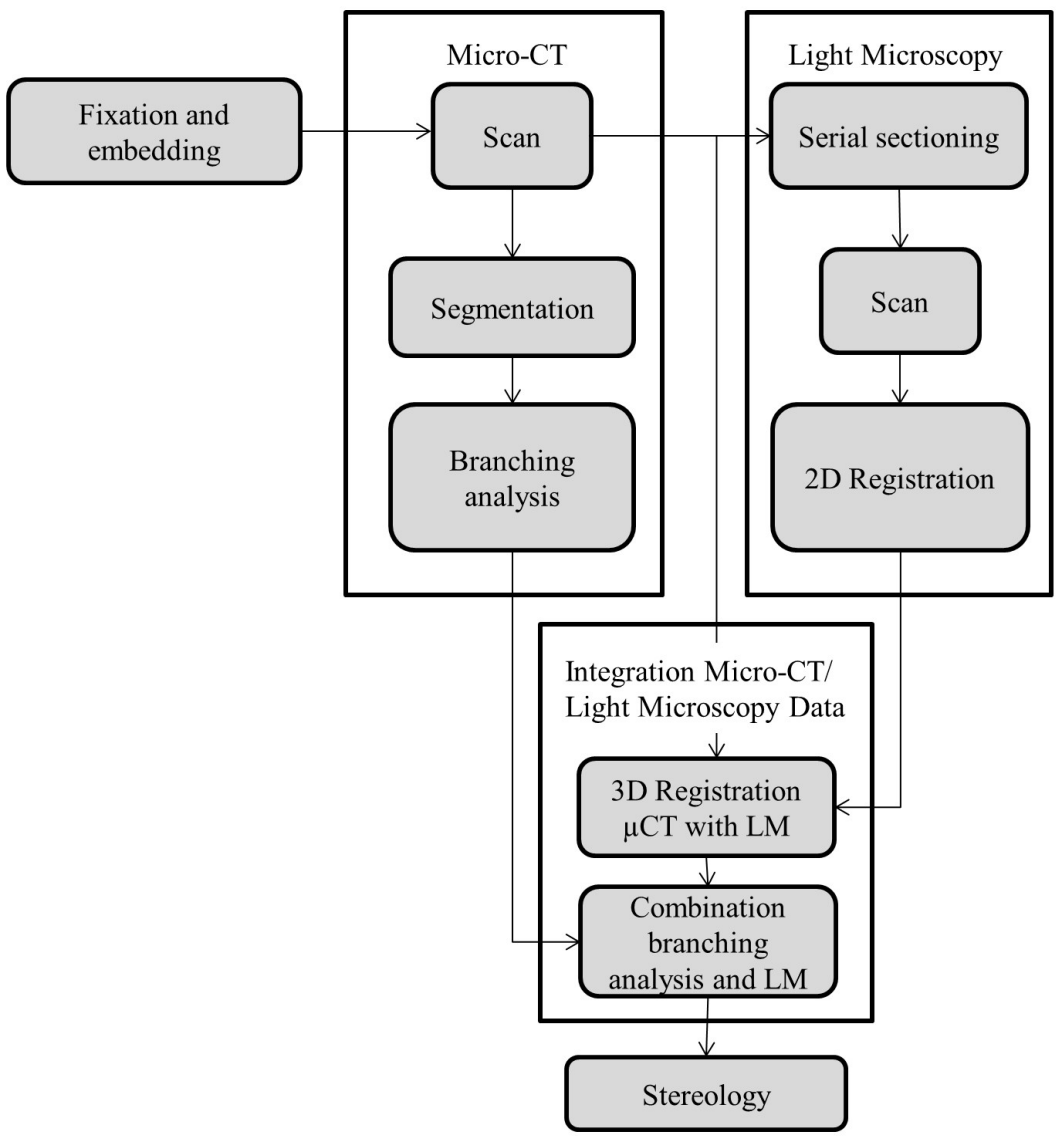
Workflow for the combination of µCT and LM data. This is a generalized version of the design by Grothausmann et al. (2021) the original image was provided under the Creative Commons license 4.0.

The workflow, as illustrated in **Figure 6**, is composed of the following steps: The organ is instilled with 4% paraformaldehyde (PFA) *in situ*, then harvested, and kept in PFA until it is contrasted for µCT as described above. It is then embedded in the embedding medium used for the following microtome cutting steps (e.g. paraffin, epoxy resin) and subsequently scanned to create an undisturbed 3D volume image. This order of preparation steps assures identical conditions for µCT and LM and thus limits the need for corrections in the registration process. On this dataset, the segmentation of the structure of interest (e.g. airway tree, pulmonary vasculature) is then conducted and further analyzed, as well as marked for e.g. branching order. In parallel, the LM-imaging is performed. To minimize the amount of material collected, while also ensuring unbiased results, a systematic uniform random sampling (SURS) (Gundersen and Jensen, 1987) procedure is followed. From a random start point, series of 24 images with a thickness between 1-5 µm (depending on the structure of interest) are collected in specific intervals (e.g. 100 µm distance). These small sample series of tissue sections are then registered within their series to each form a small sample volume. This is necessary, as single slices proved to lack enough specific features to assure an adequate registration with the lower resolution µCT images. The µCT image stack is then registered to the individual LM image stacks. The order of adjusting the µCT images to fit the LM images was chosen in order to disturb the high-resolution LM images as little as possible. Once this step has been completed, the resulting registration parameters can be applied to the segmentation image, allowing it to be overlayed on the LM images. The end result are thus LM images marked with e.g. airway orders. Morphological analyses can then be conducted, e.g. assessing cellular shape alterations in the histological images, and the results can be grouped by airway orders.

This technique has been used in BPD models to assess both pulmonary vasculature, as well as the airways in a location dependent fashion for both coarse morphology and cellular features (Labode et al., 2023; Pfleger et al., 2025) and has proven to be an invaluable tool for inter-group comparison in preclinical studies.

Reiser et al. (2025) demonstrated a pipeline incorporating even more imaging modalities, adding SRµCT and scanning electron microscopy (SEM). In addition, they scaled their process for a porcine lung, roughly equal in dimensions to a human lung. The whole organ was formalin fixed and paraffin embedded and subsequently scanned using SRµCT. Based on this first scan, regions of interest were extracted per punch biopsy and scanned in higher resolution. From these tissue cores, targeted areas where then sliced on a microtome and imaged using LM. Subareas of these slides were afterwards imaged using SEM. There was no comprehensive segmentation of e.g. airways from trachea down to alveoli, instead, limited segmentations of structures of interest were performed. The core biopsies were also not registered to the full lung scan. However, registrations between biopsy core µCT and LM and also between LM and SEM were performed. This allowed tracing of structures from the biopsy cores down to the SEM resolution level.

Salg et al. (2024) also worked on porcine tissue. While aimed at the pancreas, the general concepts of their workflow where in part inspired by and are applicable to lung research as well. In their setup, the blood vessels of the target organ were perfused with a contrast agent while still *in-situ*. The imaging pipeline then consisted of a clinical full body CT and subsequent higher resolution full organ ex-vivo scans and sub-organ level scans (µCT and SRµCT). A section of the blood vessels in a high-resolution scan were then segmented and analyzed for branching patterns. The workflow however did not include any image registrations between the different imaging modalities. So, no exact tracing of e.g. major blood vessels in the overview images into the high resolution sub-organ images was possible.

### 4.2 Integration of immunohistochemical markers

In the context of cell specific alterations, the assessment of intracellular processes is important. Previous multi step imaging pipelines however did not specifically try to include this and instead relied on basic histological stains for the purpose of the location specific assessment of cellular morphology. We thus present a novel extension to the workflow designed by Grothausmann et al. (2021), by integrating immunohistochemical markers into it. They allow the visualization of specific substances present, located within cells, on their surface, or in the extracellular space. Examples of analysis targets include club cell protein (Zuo et al., 2018) or prosurfactant apoprotein-C (SP-C) (Kim et al. (2005)) for cell identification, p21 or p53 for cell states like DNA damage (O’Reilly et al., 1998) and activated caspases 3, 8 or 9 for triggered apoptosis cascades (Tyas et al., 2000; Xiao et al., 2015).

Two distinct techniques need to be differentiated in this context. Immunohistochemistry (IHC) marks proteins by using specific antibodies binding to them. These antibodies can be visualized either by coupling them to an enzyme that catalyzes a color producing reaction or by coupling them to a fluorophore. The latter option is termed immunofluorescence (IF). To visualize the fluorophore, it needs to be excited with light of a specific wavelength and will in turn emit a different wavelength of light (Joshi and Yu, 2017). In an IF micrograph, there is thus only the marked structure visible. Times for preparation will depend on e.g. sample size, incubation times, embedding medium, etc. For this step, standard protocols can be referenced (see e.g. Hewitson and Darby, 2010; Del Valle, 2022) and will dictate the respective time requirements.

As the IHC process is compatible with histological standard stains (see e.g. Ramos-Vara, 2005), there is no inherent need to adjust the registration process between the individual LM slices or the LM image stack and the µCT volume image. Only if the labeling adds too much signal fluctuation between individual LM slices or creates signal patterns greatly differing from the µCT image, some thresholding of the image intensity may be needed.

If IF is to be used, the necessary adaptions to the workflow are more extensive. From the established method of correlative light and electron microscopy it is known, that an *OsO*_4_ treatment can reduce the intensity of a number of fluorescent proteins. Furthermore, epoxy resin used for embedding can exhibit levels of autofluorescence that might compete with the intended fluorescent signal (Kopek et al., 2017; Tanida et al., 2024). Therefore, other contrast agents, such as PTA and embedding materials like paraffin or methacrylate should be used. For the registration between µCT and IF images, either a general tissue marker, e.g. wheat germ agglutinin (WGA) for epithelial cell surface (Chazotte, 2011) or the tissue’s own autofluorescence should be used to closely match the µCT images signal distribution.

Once the registration process is completed for either method, the registered segmentation overlay can be combined with different imaging modalities as needed. For the example presented here, a left mouse lung has been stained with PTA overnight in addition to the regular histological preparation and was afterwards embedded in paraffin and scanned in a µCT machine (Phoenix Nanotom, Waygate Technologies, Germany). This is arguably a high-end device that may be prohibitively expensive for some institutes. However, previous studies (Grothausmann et al., 2021; Labode et al., 2023; Pfleger et al., 2025) successfully used scans acquired with a far less expensive desktop device (Bruker SkyScan 1272). While such devices can only handle limited sample sizes and offer reduced signal to noise ratios, they can be used in this workflow when only small animal lungs are studied. The scan time amounted to roughly two hours. If time was a concern, faster scan times with reduced image quality would have been possible as well.

The resulting volume image, with an isometric voxel size of 6.67 µm (**Figure 4a**), was segmented using a nn-Unet (Isensee et al., 2020, **Figure 4b**) that was trained on another, already segmented sample series of mouse lungs. Here, the µCT segmentation of the conducting airways has been color coded for Strahler orders (**Figure 4c**), using the Generation Analysis Toolkit (Labode and Haberthtir, 2024). Computing times are highly dependent on the hardware employed and the image sizes. Therefore, the times listed here should be interpreted as order of magnitude values. The computation steps performed for this publication were split between a Linux server (Intel Xeon CPU E5–2667 v4, 3.20 GHz with 32 cores, 1 TB RAM, NVIDIA Quadro RTX 8000 graphics card) and the Hannover Medical School High Performance Computing cluster (MHH-HPC), where similar resources were employed. Less powerful hardware could have been used as well, with the trade-off of longer computing times. All software used for the analysis is free and open source. The segmentation of the µCT volume image completed in approximately two to three hours. No manual corrections of the resulting segmentation image were necessary. The branching analysis of this dataset again ran for a similar time.

After the µCT scan, sections were cut from the tissue block and the paraffin was removed using xylol. Staining with a pro-SP-C marker was performed overnight and a WGA stain was afterwards applied for two hours. The sectioning on a microtome required about half an hour per stack of 24 slices. How many stacks are necessary depends on organ size and sampling scheme, which in turn depends on the features of interest. An example for a sampling scheme, taken from Labode et al. (2023), would be to collect 24 consecutive slices of 4 µm thickness every 300 µm. For two rabbit lungs, this resulted in 14 and 17 stacks respectively, equal to roughly one work day of preparation time each. Reducing the section thickness to 2 µm and the distance between stacks to 100 µm, increased the workload by a factor of about 1.5.

The resulting histological slides then needed to be digitized. For this task, a Zeiss Axioscan Z1 (Zeiss, Göttingen, Germany) microscopic slide scanner was used. It can perform both bright-field and fluorescent imaging, processes up to 100 slides at a time, and can handle multiple organ slices on one glass slide (depending on organ size, two to three slices will fit on one slide). Scan times will mainly depend on the size of the slices and the magnification setting. The scan time for IF slices will depend on the number of markers employed, i.e. two markers require two scanning cycles with different wavelengths of light, equaling double the time required for a comparable bright-field image acquisition. The scan process is fully autonomous, and a rough estimate for the scan duration is overnight, i.e. the scanner can be loaded with the slides in the afternoon and unloaded the next morning. Simpler camera/microscope setups can be used as well, with the trade-off of an increased manual workload. Due to the high sensitivity of the IF stains to external stressors, the slides should be kept away from light sources and may be refrigerated until imaging. The time between staining and imaging should be kept to a minimum, i.e. hours rather than days.

**Figure 7:**
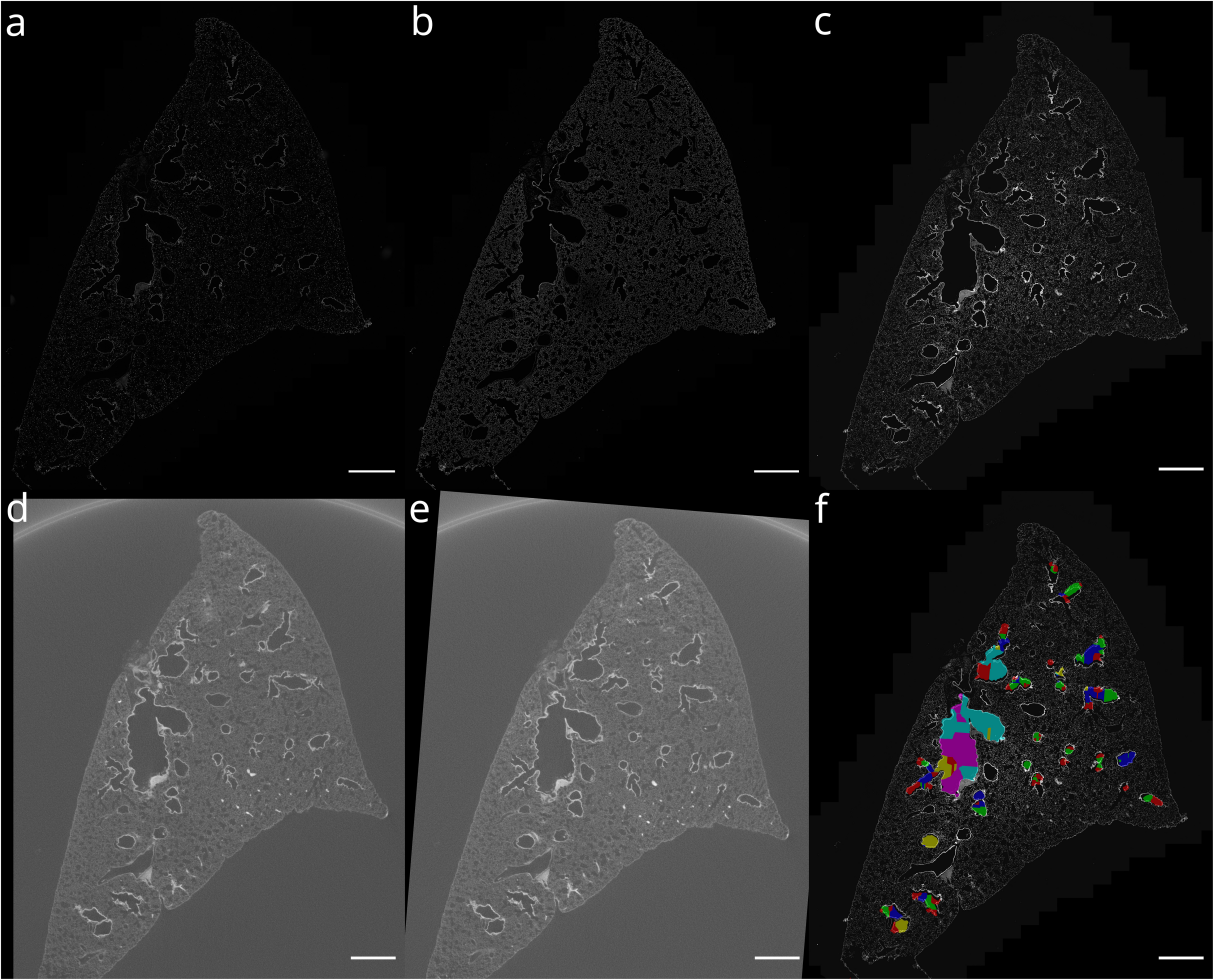
Steps of the registration process. While the result is not perfect (mostly due to the damages the IF slice, sustained in the cutting and preparation process), the segmentation overlay allows for a clear order attribution. **(a)** Pro-SPC channel of the IF image. **(b)** WGA channel of the IF image. **(c)** Autofluorescence channel of the IF image. Chosen for the registration, as its signal pattern most closely resembles that of the µCT image shown in **(d)**. **(d)** µCT image stack translated laterally, z-position approximately matching that of the IF image in **(c)**. This is the starting point for the registration process. **(e)** Registration result: The µCT image stack has been rotated in all three dimensions to achieve the highest overlap of features with the IF image. **(f)** The registration parameters resulting from **(e)** have been applied to the segmentation image (see **Figures 4c,d**). The result has been overlayed on the IF image **(c)**. Scale bars = 1000 µm.

**Figure 8:**
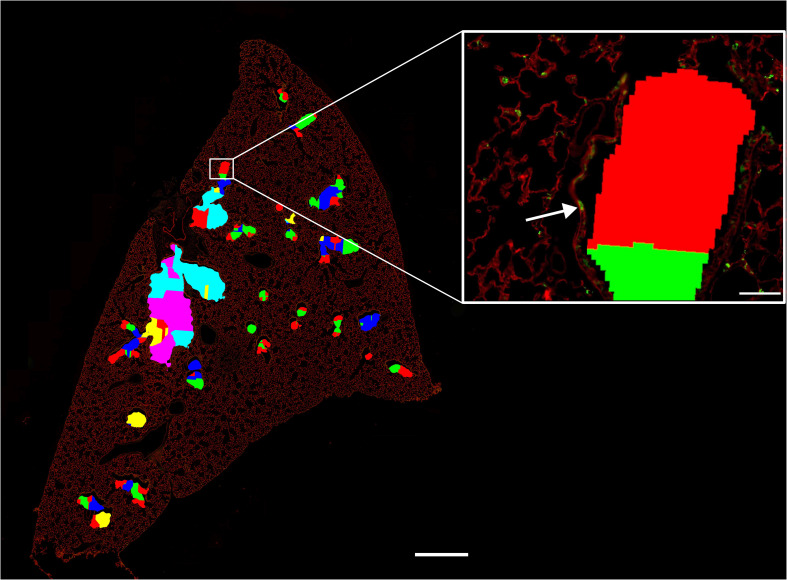
WGA (red) and pro-SP-C (green) channels of the IF image from **Figures 7a–c, f**, combined with the segmentation image. Scale bar = 1000 µm. Inset: The arrow points at a pro-SP-C positive cell in the airway. In a study setting, this would present a counting event for order 1. Scale bar = 50 µm.

**Figure 9:**
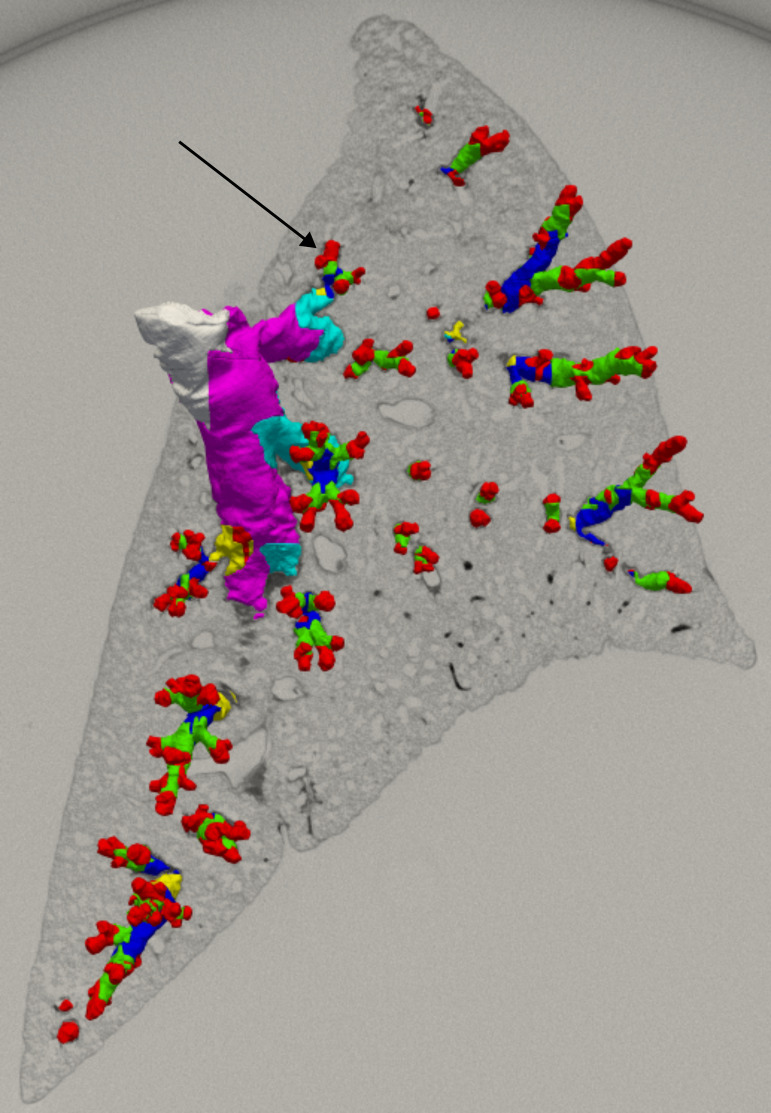
µCT image slices combined with the 3D segmentation image. The arrow points at the region visible in the inset of **Figure 8**.

In the following registration process, using the software elastix (Klein et al., 2010; Shamonin, 2013), the µCT volume image was transformed to achieve the highest possible fit with the IF image. **Figure 7** illustrates the individual steps. Using the registration parameters resulting from this operation, the segmentation image could be adjusted in the same way and was then superimposed on top of the corresponding IF-section. The results are visualized in **Figure 8**, additional 3D context from the µCT is supplied in **Figure 9**. Image registration between the individual slices of a single stack again required two to three hours per stack. Here, in some cases, the result was deemed unsatisfactory and the images were adjusted (they were e.g. manually rotated, artifacts were digitally removed from the images or they were excluded if damages sustained in the cutting process were too severe for registration). In such a case, the registration job for the stack had to be rerun after the adjustments. The last preparation task was the registration between µCT and LM/IF stack. Here, the time per stack was roughly equal to the previous registration task. In some cases, the spatial position of the stack in relation to the full organ volume needed adjustment and repeated execution. All computational jobs required little to no manual interaction at runtime. They could be scripted to run in an efficient manner and quality control could be performed at opportune times. Thus, the manual work spent on these tasks was only a fraction of the compute times.

In the resulting images (**Figures 7**, **8**), the WGA stain marks all cell borders, while the pro-SP-C marker highlights specific cells containing SP-C. Labeled cells in the airways are broncho-alveolar stem cells that are characteristic for the transitional zone between bronchiole and alveoli. In the alveolar region, type two pneumocytes are marked (see Eenjes et al., 2022).In a study with the goal to quantify the distribution of broncho-alveolar stem cells within the airways, marked cells would be counted per airway section (see **Figure 8** inset). The time required for this final analysis step of the pipeline, is highly dependent on the study goal. If e.g. the location of a rare cell type, marked with IF, along the segmented airway is to be collected, mere hours may be required. If on the other hand a representative collection of cell types and their dimensions at different airway branching orders is to be acquired, the task can require weeks of cell identification and measuring. In the future, automation of feature recognition might replace these manual analysis steps.

While a marker for cell death would have been preferable in the context of this review, such a demonstration would have required fresh sample material from a lung injury model. Due to the ethical concerns raised by such models, appropriate material is scarce and was not available at the time of writing. While normal cell turnover and thus cell death is present in adult lungs, rates are so low, that witnessing such an event in the sampled region is a chance encounter. Therefore, the pro-SP-C marker was used for this demonstration, as it is a well-established and robust marker for proteins, that are present in a physiological state of the lung. Nevertheless, the demonstration presented here serves as a proof of concept to illustrate the possibility to integrate IF imaging into the existing workflow. In an appropriate model setting, this procedure can be performed in an analogous fashion with different markers, such as one of the well-established cleaved caspase 3 or 9 markers for apoptosis (see e.g. Bern et al., 2010; Kuwano et al., 2002; Petrache et al., 2006; Wang et al., 2017).

When designing a study using the workflow proposed above, researchers can fine tune the setup to their requirements. The most basic decision however, is the choice of the immunohistochemical marking approach. Hence, some advantages and disadvantages to consider will be discussed here. IHC is easier to incorporate into the imaging process, as it requires only regular LM images. It also allows for simultaneous assessment of tissue morphology using the standard stain and histochemical processes marked by the enzyme. This facilitates the direct correlation of morphological and chemical alterations in the tissue. Stains are also more robust against e.g. photobleaching. IF on the other hand is more delicate, in that it fades rapidly and can be degraded by light exposure, e.g. in the imaging process. Thus, samples may require a quick succession of staining and imaging, special storage conditions and may not allow for repeat imaging. Additional image modalities for the registration process (i.e. a general marker in addition to the specific protein marker) can be necessary as well. This implies additional staining steps, as well as imaging time. Nevertheless, the IF approach excels, when only small amounts of the target protein are present, that might be hard to recognize in a basic IHC application. Additionally, multiple markers in different wavelength can be employed simultaneously, allowing for the detection of different substances in the same tissue section.

A main feature of the workflow presented here, is the reference of findings by their position along the airway tree. This element is important for the translation of study results between lungs of animals and humans. How such a translation can be performed, is highly dependent on the animal used and the structures of interest. Therefore, only some general aspects, examples and limitations, not an exhaustive description, can be provided here. One main factor to consider is the branching pattern of the airways. In human lungs, they branch out in a relatively symmetrical fashion, with each airway branch producing two morphologically similar daughter branches upon division (Weibel, 2009). Common lab animals on the other hand exhibit a highly asymmetrical, so called monopodial, branching pattern, characterized by a long central airway with significantly smaller lateral branches (Phalen and Oldham, 1983). This asymmetry is the reason, why a generation attribution is not practical in these lungs (see Labode et al., 2022). Therefore, when e.g. comparing coarse morphology, the airway sections of order six and seven in the mouse lung, as displayed in **Figures 4c, d**, are the structures most closely resembling the larger bronchi (first few generations) of the human lung.

However, beyond the difference in branching pattern, further structural disparities are present, that will influence the translation between species. For example, cartilage or mucus glands, as found in the human bronchi, are not present in mice, rats or rabbits. Furthermore, goblet cells e.g. in the rabbit are only found in the most proximal airways (Irvin and Bates, 2003; Hyde et al., 2006; Plopper et al., 1983). So, when e.g. studying mucociliary clearance, the larger conducting airways of the rabbit lung more closely resemble the situation in the larger bronchioles of the human lung, where glands are absent and goblet cells are responsible for mucus production until they get replaced by club cells in the distal bronchioles with the transition to the surfactant system (see e.g. Rogers, 2003). In that case, matching the higher orders of the animal lung to the bronchiole generations of the human lung may be more suitable, while a model of the bronchi would require another animal species.

When studying the gas exchange region, it needs to be taken into account, that mice or rabbits do not, or only in rare cases, possess respiratory bronchioles (Hyde et al., 2006; Kamaruzaman et al., 2013), while they make up about three generations in the human lung (Weibel, 2009). So, while structural comparisons for alveolar ducts and alveolar sacs could be drawn between species, there is no correlate for the respiratory bronchioles in the mouse model and thus no transfer possible.

Finally, a discussion of workflow limitations is in order. These inherently include the limitations of LM and µCT, which have been listed above. In their combination however, additional aspects, such as the achievable combined resolutions need to be considered. The extent of the segmentation markings extracted from the µCT image, used for reference purposes, is limited by the µCT resolution. In small animals, such as mice or rabbits, the conducting airways can be segmented without issue. The gas exchange region however cannot be sufficiently resolved. Previous BPD-studies for example measured mean alveolar septum thicknesses below 10 µm in the mouse (Appuhn et al., 2021), too small to be adequately resolved in the µCT image. This is demonstrated in **Figure 5**. If the vasculature is the segmentation target, arteries and arterioles down to about 30 µm in diameter can be segmented (Labode et al., 2022). This would apply to the venous side analogously. Precapillary and capillary vessels would be beyond reach. In the LM images, on which the segmentation images are superimposed and which are used for the structural analysis, far higher resolution levels can be achieved. In theory, a resolution of about 0.2 µm for visible light is possible (Abbe, 1873). As the maximum resolution is dependent on the wavelength of light that is used, IF microscopy using ultraviolet light can further lower this boundary. In practice, this value will not be reached, with the achievable resolution depending on e.g. the numerical aperture of the objective, auto focus performance and camera resolution. See e.g. Geubbelmans et al. (2024) for implications of different microscopic scan settings. Nevertheless, results are fully sufficient for the study of sub-cellular features. Examples include the counting cell nuclei or the measurement of ciliary length in rabbit airways by Pfleger et al. (2025). Due to their lower resolution, the borders of the segmentation markings can appear rough and boxy when placed over the higher resolution LM or IF images (see inset in **Figure 8**), but are by far sufficient for orientation.

The sequential use of both imaging techniques is also susceptible to changes to the tissue between the µCT imaging and LM imaging step, as those hinder the registration between both imaging modalities or in the creation of the LM image stacks. However, while a low number of slices or stacks might have to be excluded from analysis, a representative number of stacks has been reached by a high margin in the studies where this procedure has been employed in the past. Another obstacle that this approach can encounter, is airway or vessel collapse due to low instillation/perfusion pressure or blockage by an artifact in the preparation process. This would make a segmentation of the affected structure unfeasible. The reference of the occurrence of features by their location along the airway- or vessel tree, that is central to the presented method, would thus be prevented. Care must therefore be taken in the initial organ preparation steps.

## 5 Summary and outlook

Both LM and µCT are important tools for studying the lung. Nevertheless, for the location specific analysis of cellular alterations, a combination of both imaging modalities, delivers the most conclusive results. Several studies have presented multi-step imaging pipelines, that successfully integrated different imaging modalities, to combine high resolution images with full organ spatial references. It could further be demonstrated, that such a workflow can be adapted to complex research questions, where not only morphology is important, but also the identification of intracellular processes using IHC or IF. This method can therefore serve as an important tool for further researching the role of cell death in BPD. Its use in future studies may provide information about how the location of acute cell stress and cell death relates to the location of chronic changes that manifest and persist in the lung tissue later in life. The identification of main drivers, i.e. intracellular processes that show the highest correlation with morphological changes may be possible, in turn identifying potential targets for treatment approaches. It can support addressing a main issue of preclinical models of lung disease, by aiding contextualized data collection and advance the translation of results to the morphology of the human lung.

Broadening the view, the general research goal should be to combine knowledge of pathology manifestation, gathered from both clinical and preclinical research, to a conclusive model of what drives disease progression at which time and at what location. An important aspect that needs further research is the translation of the site of pathological changes in preclinical animal models, where lung morphology (e.g. a monopodial branching pattern) and airway characteristics (no bronchus with cartilage or glands is present in mice or rabbits) differ from the human lung they are supposed to represent.

While this review focused on lung pathology research, it should nevertheless be mentioned, that the methods of location specific histology can be applied to other research questions, such as treatment strategies, as well. Once targets for pharmaceutical intervention are identified, a way for optimal drug delivery needs to be formulated. Here, the discussed workflow could be essential to identify e.g. particle deposition patterns of inhaled pharmaceuticals in respect to the branching morphology of the targeted lung.

With imaging technology further advancing and enabling increasingly deeper and more comprehensive insights into the lung, techniques that allow locating that information within the structure of the organ and facilitate the identification of representative sampling locations, increasingly gain in importance.
